# Leveraging developmental neurotoxicity data in zebrafish embryos through the use of artificial intelligence methods

**DOI:** 10.3389/ftox.2026.1789691

**Published:** 2026-04-01

**Authors:** Harm J. Heusinkveld, Ellen V. S. Hessel, Edwin P. Zwart, Eric R. Gremmer, Jeroen L. A. Pennings

**Affiliations:** Centre for Health Protection, National Institute for Public Health and the Environment (RIVM), Bilthoven, Netherlands

**Keywords:** *Danio rerio*, developmental neurotoxicity, machine learning, pharmaceutical, ZebraBox

## Abstract

**Introduction:**

The zebrafish is a well-known whole organism model to study developmental effects of chemical exposure, including developmental neurotoxicity (DNT). One method used to screen for DNT effects is the zebrafish light-dark transition test (LDT), which measures locomotory behavior under alternating light and dark conditions. Because the LDT is relatively new, experimental protocols and data analysis tools for this assay are still evolving. To advance our knowledge on potential applications of the LDT, we explored using artificial intelligence (AI) methods to distinguish between zebrafish exposed to vehicle controls or pharmaceuticals (fluoxetine, paroxetine, carbamazepine, phenytoin) on 5, 10 or 14 days post-fertilization (dpf).

**Methods:**

AI methods were trained to distinguish control versus exposed zebrafish behavior and then applied to assign fish to either group, using five-fold cross-validation. This was done with four different AI methods that differ in nature and complexity, namely Generalized Linear Model (GLM), Random Forest (RF), Gradient Boosting Machine (GBM), and Deep Learning (DL).

**Results:**

Average prediction accuracy increased from 67% at 5 dpf to 76% at 10 and 14 dpf upon continuous exposure. For fish analyzed at 14 dpf but exposed for shorter durations, we found DNT effects clearly persistent for the antidepressants fluoxetine and paroxetine, but less clearly for the anticonvulsants carbamazepine and phenytoin. The AI methods GLM, RF and DL showed comparable performance, whereas GBM accuracies were lower.

**Discussion:**

Compared to conventional univariate analysis, AI appears more sensitive in detecting DNT effects. Overall, this shows the potential of implementing AI methods in DNT screening of chemicals and further development of this approach.

## 1 Introduction

In the last decade, there has been a notable increase in the number of scientific studies into developmental neurotoxicity (DNT). Partially, this was driven by claims that prevalence rates of autism spectrum disorder and attention-deficit hyperactivity disorder seem to be increasing worldwide (Landrigan et al., 2012). Moreover, there is increasing awareness that early exposure to environmental chemicals can adversely affect fetal brain development and later neurological health (Grandjean and Landrigan, 2014). Additionally, advances in genomics, neuroimaging, and stem cell research have opened new avenues for studying developmental neurotoxicity, enabling more in-depth and detailed analyses (Kanton et al., 2019; Ku and Asuri, 2024; Volkow et al., 2024). However, animal studies into DNT are complicated by the fact that neural development is a complex process involving many different events, each with its own time frame. As a result, each event throughout brain development may have a different window of vulnerability to toxicant exposure (Rice and Barone, 2000; Ingber and Pohl, 2016; Heyer and Meredith, 2017).

The zebrafish (*Danio rerio*) is a well-established whole-organism model for DNT screening. The zebrafish nervous system, including the brain, shows organizational homology with humans and other vertebrates, including the neurotransmitter system, the blood–brain barrier, and the thyroid system (Jeong et al., 2008; Kozol et al., 2016). Moreover, the small size and rapid development of zebrafish larvae make them suitable for medium- to high-throughput screening methods (Collins et al., 2024). This makes them a useful model for studying the effects of chemical exposure in whole organisms, including gene expression, neural cell and tissue development, and brain functioning and neurobehavior, from the perspective of improving human health (Nishimura et al., 2015; Samrani et al., 2023).

Several tools can be used to assess the effects of chemical exposure on DNT in zebrafish (de Esch et al., 2012). One commonly used method, recently investigated by the OECD and used by various laboratories, is the light-dark transition (LDT) test (MacPhail et al., 2009). This test uses an automated video tracking system to monitor the behavioral responses of freely swimming zebrafish under alternating light and dark conditions and to assess how locomotor activity changes upon the switch from light to dark (and *vice versa*). Normal behavior consists of low activity under light conditions and increased activity upon switching to dark conditions (MacPhail et al., 2009). Aspects of this behavior (e.g., time spent in activity or average speed) are used as a phenotypic marker for (developmental) neurotoxicity, and the effects hereon of exposure to (suspected) toxicants (MacPhail et al., 2009; Atzei et al., 2021; Havermans et al., 2021; Haigis et al., 2022; Hessel et al., 2022). Although locomotor activity and its changes can be perturbed by neurological or non-neurological factors, the LDT is considered a sensitive endpoint for DNT assessment because it relies on the integrity of brain function, nervous system development, and visual pathways. Hence, the behavioral endpoint(s) can be used to screen for DNT effects of chemical exposure based on the function of the brain represented by its neurobehavior (Bilotta et al., 2002).

To date, there is no standardized protocol for performing an LDT, leading to variation between laboratories in how the LDT is conducted (MacPhail et al., 2009; Atzei et al., 2021; Hill et al., 2023), as well as in how data are reported and analyzed. Several studies have explored ways to improve data analysis (Haigis et al., 2022; Hill et al., 2023; Rowson et al., 2025). In many cases, the analysis focused on one or several parameters that are considered sensitive to DNT perturbation, such as time in activity (Atzei et al., 2021) or distance traveled (Haigis et al., 2022) during light and/or dark conditions. However, it seems reasonable to expect that there can be additional information available not only in other parameters, but also in how various parameters change over time. Moreover, because the various parameters each describe an aspect of the same swimming behavior, some parameters may act in concert. As a result, several smaller changes may add up to an overall meaningful DNT effect. Reasons why researchers generally refrain from analyzing their data in more detail are that it would make data analysis more time-consuming and more complex to interpret. Moreover, statistical analyses involving a larger number of parameters would need to account for the chance of false-positive findings.

In this article, we explore the use of artificial intelligence (AI) or machine learning (ML) tools to distinguish between treated versus control zebrafish and to assess their predictive performance on LDT data as an overall measure for the presence of DNT effect. For this purpose, we used four different methods that differ in nature and complexity. The generalized linear model (GLM) can be considered the least complex of the four methods and uses logistic regression for binary classification. Random forest (RF) works by building a set of independent decision trees and combining their results. Each tree uses a number of questions to classify samples into one of the two classes, and the most common prediction for the tree collective becomes the RF prediction output. Gradient boosting machine (GBM) also uses prediction trees but differs from RF in several ways. Most importantly, the trees are not created independently. Rather, they are created sequentially, and each tree is created to improve the prior one by reducing the difference between the predicted class probabilities and the true class probability (i.e., 0 or 1). Deep learning (DL) is based on an artificial neural network that “learns” by iterative analysis of training samples and gradually optimizing parameters during the process. Neural networks can be applied to a wide range of tasks and represent the most advanced AI method among the four used in this study. Additional details on how these methods work and differ from each other can be found in Orcales et al. (2024). In addition to an evaluation of their value for the detection of DNT effects, using four inherently different types of AI/ML also allows us to compare these tools both in terms of predictive performance and regarding method-specific strengths and pitfalls.

## 2 Methods

### 2.1 Pharmaceuticals

The test pharmaceuticals fluoxetine hydrochloride (FLX, CAS 54910-89-3; cat. no. F132; ≥98% purity), paroxetine hydrochloride (PAX, 110,429-35-1; cat. no. 1500218; pharmaceutical standard), carbamazepine (CBZ, CAS 298-46-4; cat. no. C4024; ≥98% purity), and phenytoin (PHT, CAS 57-41-0; cat. no. P1290000; pharmaceutical standard) were obtained from Sigma-Aldrich (St. Louis, MO, USA). Immediately prior to exposure, stock solutions of all pharmaceuticals were prepared using dimethyl sulfoxide (DMSO; Merck, Darmstadt, Germany) at 1,000× concentration. These stocks were further diluted in embryo medium (containing NaHCO_3_ (100 mg/L), KHCO_3_ (20 mg/L), CaCl_2_∙2H_2_O (200 mg/L), and MgSO_4_∙7H_2_O (180 mg/L) in demineralized water), resulting in a final concentration of 0.1% DMSO in all conditions. Embryos under control conditions were exposed to DMSO only (final concentration 0.1%).

### 2.2 Zebrafish maintenance and exposure

All procedures involving fish older than 5 dpf were scientifically and ethically reviewed and approved according to Dutch regulation (license CCD-2016-0052).

Wild-type (AB strain) zebrafish were maintained and bred in an automatic ZebTec flow-through system (Tecniplast S.p.A, Buguggiate, Italy) in the National Institute for Public Health and the Environment (RIVM) facility. In the facility, pH was maintained at 7.5 ± 0.5, temperature at 27.5 °C ± 0.5 °C, and conductivity at 500 ± 100 μS with a photoperiod set at a light/dark cycle of 14/10 h. Adult zebrafish were fed three times a day, twice with SDS (Special Diet Services, Tecnilab-BMI BV, Someren, the Netherlands) 100 (Cat. 824856), 200 (Cat. 824862), 300 (Cat. 824867) or small granules (Cat. 824876) (depending on age), and once with frozen *Artemia* (Superfish). For breeding purposes, females were separated into a 3.5-L tank and fed frozen *Artemia* three times a day for a period of 4 days to stimulate egg production. The evening prior to mating, four females and four males were joined in a 1.7 L sloped breeding tank. Spawning was triggered by the onset of light, and eggs were collected within 30 min after spawning. Eggs were transferred to Petri dishes and successfully fertilized eggs of good quality (symmetrical development; ≤10% coagulated eggs in total), as evaluated under a stereomicroscope (Leica M8), were kept for further use.

To determine non-embryotoxic concentration ranges for subsequent behavior experiments, development and teratology of exposed embryos were assessed at both 3 dpf and 5 dpf under a stereomicroscope in a zebrafish embryo toxicity test (ZFET), as previously described (Hermsen et al., 2011). In brief, eggs at the cleavage stage were exposed to one of the four test pharmaceuticals or DMSO in 24-well plates containing one egg in 2 mL embryo medium (Staal et al., 2018). Embryo development was scored on the following endpoints: tail detachment, somite formation, eye development, movement of the embryo, heartbeat, blood circulation, embryo pigmentation, pectoral fin, protruding mouth, and hatching. Next, teratological effects were recorded based on the presence of pericardial edema, yolk sac edema, eye edema, head malformation, absence/malformation of sacculi/otoliths, tail malformations, heart malformations, modified chorda structure, scoliosis, rachischisis, and yolk deformation. These developmental and teratological scores were used to determine the non-embryotoxic concentration range for subsequent behavioral analysis.

For the behavioral assessment, zebrafish embryos were exposed in a 6-well plate (20 eggs per concentration and solvent control) containing 5 mL of test medium and kept in an incubator at 27.5 °C ± 0.5 °C. Concentration–response data were obtained for all exposed embryos at 14 dpf. Based on these data, benchmark dose modeling (BMD) was applied using the benchmark method in the PROAST dose–response modeling tool in R (https://proastweb.rivm.nl) (Slob, 2002; Hardy et al., 2017) to obtain the dose that induced a 20% change in the average activity during the dark period (critical effect dose at 20%; CED20). This dose was used in further experimentation, and embryos were exposed to 0.16 µM fluoxetine, 0.087 µM paroxetine, 102 µM carbamazepine, 106 µM phenytoin, or embryo medium (vehicle) control. For this study, five exposure scenarios were used: LDT analysis at 5 dpf, 10 dpf, or 14 dpf after continuous exposure, or LDT analysis at 14 dpf after exposure during 0–10 dpf or 0–5 dpf. In all experiments, exposures started at ∼2 h post-fertilization (hpf), and medium was refreshed at 5 dpf, 7 dpf, 10 dpf, and 12 dpf.

### 2.3 Zebrafish locomotion

Swimming locomotory behavior data were determined using a light-dark transition test (LDT) using a ZebraBox (Viewpoint, Lyon, France) following a previously described protocol (Atzei et al., 2021). In brief, 12 larvae per concentration were transferred along with 300 µL of test medium to a round-bottom 96-well plate (5 dpf; 1 larva per well) or 2 mL of test medium per well in a 24-well plate (10 dpf and 14 dpf). Following an acclimatization period of 30 min in the light, free-swimming activity was recorded in the ZebraBox during three cycles of alternating light and dark phases, each phase lasting 10 min, so that the key locomotory behavioral prompts were three light-dark transitions and two dark-light transitions. Recorded movies were analyzed using ZebraLab Quantization software (Viewpoint, Lyon, France) with a sensitivity setting of 20 and thresholds of 10 (burst) and 1 (freezing). This resulted in locomotor data for a total of 13 parameters (Table 1) per minute over a 60-min period. Data from several experiments were collected and merged into an MS Excel file.

**TABLE 1:** ZebraBox parameters.

Parameter	Full name
inadur	Time inactive
inadist	Distance while inactive
inaspd	Speed while inactive
smldur	Time a little active
smldist	Distance while a little active
smlspd	Speed while a little active
lardur	Time very active
lardist	Distance while very active
larspd	Speed while very active
dist	Distance covered
distb	Distance covered in bursts
dur	Time active
durb	Time active in bursts

### 2.4 Data analysis

Data were analyzed in R statistical software (version 4.5.0) using the reshape (version 0.8.10) and h2o (version 3.44.0.3) packages.

Zebrafish locomotory data were reformatted so that for each zebrafish larva, a matrix of 13 parameters over 60 min was turned into a vector of length 780 (=13 × 60). Thus, for AI/ML, we had 780 parameters, each defined as the combination of one locomotory parameter (Table 1) during 1 minute of the 60-min analysis time (for example, distance covered during minute 40). No additional normalization or scaling was done at this stage.

Data for pharmaceutical-exposed and control larvae (n = 35–36 each) were compared by univariate statistical analysis. Data for each of the 780 parameters were compared between the two groups using a Mann–Whitney U test. Raw p-values were adjusted for multiple testing by using the Bonferroni correction. Parameters with adjusted p-values <0.05 were considered significant.

Next, data for pharmaceutical-exposed larvae were compared to controls using four AI/ML tools in the h2o package, namely, the GLM, RF, GBM, and DL functions. More information on this package and the functions used is available at the h2o R package vignette (H2o.Ai, 2025) and the functions h2o.glm(), h2o.randomForest(), h2o.gbm(), and h2o.deeplearning() described therein. The default settings were used for each of these AI/ML tools. Classification was performed twice, once with control samples coded as class 1 and exposed as class 2, and once the other way round (to balance out algorithmic preferences for either value), both times using fivefold cross-validation. This kind of cross-validation divides the data into five parts, trains the algorithm on four parts of the data, uses this to predict the class for the remaining part (also known as the test set), and repeats this five times so that every part of the data gets used as the test set once. Classification output was combined to calculate overall accuracy values, as well as the sensitivity (recall), specificity, positive predictive value (precision), and negative predictive value. The univariate and AI/ML approaches were also used to pairwise compare the 14-day control groups for each of the four pharmaceuticals.

Finally, we applied the four algorithms to random(ized) data. This was done by (a) using 100 data sets of the same size and format as a two-group LDT comparison but filled with uniformly distributed random data, and (b) repeated class label permutations on exposure-control data comparison data sets, giving a total of 100 or more randomized data sets. For both approaches, the combined output per algorithm was used to determine the baseline accuracy and confidence intervals.

An R script containing the AI/ML methods described is available at GitHub, under https://github.com/jlapennings/zebrain.

## 3 Results

We first performed univariate statistical analysis on the 780 parameters. The number of statistically significant parameters is shown in Table 2. Generally, the number of significant parameters is low or zero for the zebrafish that were analyzed on day 5 after continuous exposure (Table 2, first data column). The numbers were higher for fish analyzed on days 10 and 14 after continuous exposure (second and third data columns), which suggests that the DNT effects are not yet fully present on day 5. For fish analyzed on day 14 but exposed from day 0–5 or 0–10, the number of significant parameters is reduced, in some cases even to zero. This is especially the case for carbamazepine and phenytoin, both anticonvulsants, and less so for the antidepressants fluoxetine and paroxetine. This indicates that at least some of the anticonvulsant-induced effects are reversible.

**TABLE 2:** Number of significant univariate parameters.

Pharmaceutical	Day 5, exposed d0–d5	Day 10, exposed d0–d10	Day 14, exposed d0–d14	Day 14, exposed d0–d10	Day 14, exposed d0–d5
Fluoxetine	0	325	208	323	0
Paroxetine	20	111	102	4	12
Carbamazepine	0	90	166	0	0
Phenytoin	40	155	119	0	1

Parameters were compared between pharmaceutical-exposed and control zebrafish (n = 35–36 per group) by a Mann–Whitney U test. Raw p-values were adjusted for multiple testing by Bonferroni correction. Adjusted p-values <0.05 were considered significant.

**FIGURE 1:**
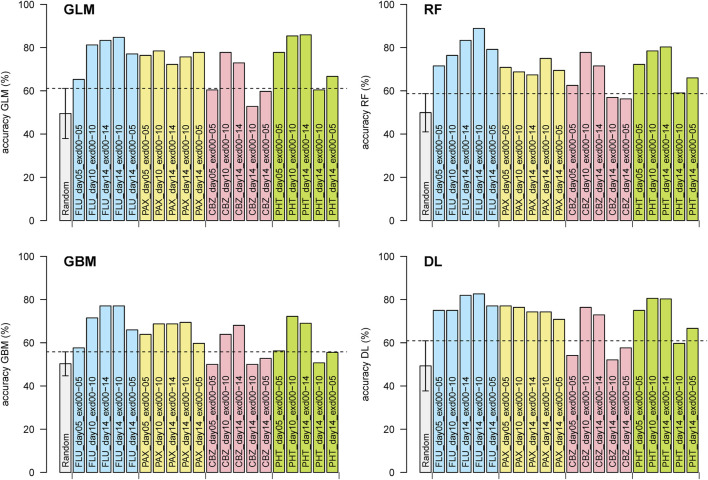
Predictive performance (cross-validation accuracies) of the h2o package classifications. Labels indicate pharmaceutical, measurement day, and exposure duration. RF, random forest; GLM, generalized linear model; GBM, gradient boosting machine; DL, deep learning. FLU, fluoxetine; PAX, paroxetine; CBZ, carbamazepine; PHT, phenytoin. Error bars indicate the 95% confidence interval, and dashed lines indicate the upper limits of the 95% confidence intervals for label-permutated data.

**FIGURE 2:**
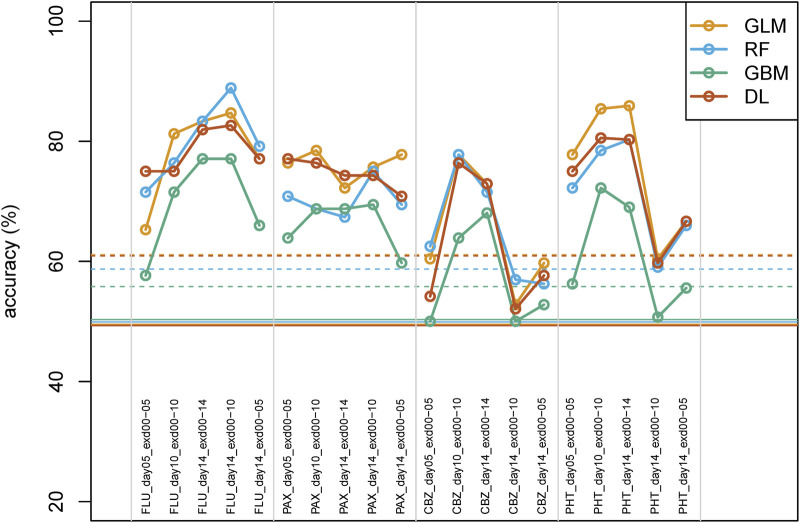
Predictive performance (cross-validation accuracies) of the h2o AI/ML tools. RF, random forest; GLM, generalized linear model; GBM, gradient boosting machine; DL, deep learning. The horizontal solid and dashed lines indicate the average and the upper limit of the 95% confidence interval, respectively, for each method, based on label-permutated data.

AI/ML classification on swimming locomotory data of pharmaceutical-exposed versus control larvae (Figures 1, 2) resulted in prediction accuracy values that averaged at 76% for continuous exposure to days 10 or 14 and at 67% on day 5. This is in agreement with the lower number of significant univariate parameters on day 5 (Table 2). In addition, in line with the univariate results, for fish analyzed on day 14 but exposed for shorter durations, the prediction accuracy for anticonvulsants is reduced, with more pronounced changes for the antidepressants.

Aligning the accuracies for the various algorithms across the experimental group comparisons indicates that GLM, RF, and DL follow a globally similar pattern (Figure 2), whereas GBM follows a qualitatively similar pattern but with globally lower accuracies than the other three methods. Similar findings were obtained for the sensitivity, specificity, positive predictive value, and negative predictive value (Supplementary Table S1).

Because AI/ML tools are developed to get the most discriminatory power from a data set, it can be relevant to know at which point the data set shows better (or similar) class discrimination than for random(ized) data. We repeatedly determined the prediction accuracy for all four of the h2o AI/ML tools on sets of random data. All methods had a comparable performance for accuracy (GLM 49.6% ± 3.6%, RF 49.6% ± 4.3%, GBM 49.8% ± 1.7%, DL 50.7% ± 4.3%). Additionally, we determined prediction accuracies upon repeatedly permutating class labels. Results were also comparable for this approach (GLM 49.5% ± 5.9%, RF 49.9% ± 4.5%, GBM 50.3% ± 2.8%, DL 49.3% ± 5.9%), albeit with larger standard deviations than for fully random data. The corresponding 95% confidence intervals for label-permutated data are shown in Figure 1. The overall average for label-permutated data was 49.7% ± 5.0% (standard deviation calculated as the square root of the average variance), corresponding to an upper 95% confidence limit of 59.5% as a threshold for significant class discrimination (Figure 2).

Finally, we applied AI/ML tools to compare the 14-dpf unexposed control groups to the groups exposed to the various pharmaceuticals. For all pairwise combinations, AI/ML tools were able to distinguish control groups significantly better than could be expected for random data (Table 3). In four of these cases, univariate analysis also identified statistically significant parameters; in two cases (PAX-PHT and CBZ-PHT), AI/ML proved more sensitive in distinguishing control groups.

**TABLE 3:** Comparisons between control groups on day 14.

Method	FLU-PAX	FLU-CBZ	FLU-PHT	PAX-CBZ	PAX-PHT	CBZ-PHT
NSUP	15	2	3	2	0	0
GLM accuracy (%)	79.9	72.2	68.1	82.6	67.4	63.9
RF accuracy (%)	75.7	70.8	73.6	77.1	75.7	58.3
GBM accuracy (%)	63.2	56.3	59.0	59.7	63.2	54.9
DL accuracy (%)	72.2	75.7	79.2	76.4	70.8	59.0

NSUP, number of significant univariate parameters; RF, random forest; GLM, generalized linear model; GBM, gradient boosting machine; DL, deep learning. FLU, fluoxetine; PAX, paroxetine; CBZ, carbamazepine; PHT, phenytoin.

## 4 Discussion

In recent years, the zebrafish LDT has been increasingly used to study developmental neurotoxic effects of compound exposure. Many of these recent articles evaluated effects of neuropharmaceuticals (Leuthold et al., 2019; Atzei et al., 2021; Havermans et al., 2021; Gundlach et al., 2022; Zhou et al., 2023; Gericke et al., 2024; Liu et al., 2024; Knapp et al., 2025; Rowson et al., 2025). Additionally, several studies have focused on pesticides (Leuthold et al., 2019; Konemann et al., 2022; Rowson et al., 2025), other chemicals (Pipal et al., 2020; Glazer and Brennan, 2021; Okeke et al., 2022; Hu et al., 2024; Pretorius et al., 2024; Qiu et al., 2024), or environmental factors such as salinity (Scarlett et al., 2022). Furthermore, the LDT has been applied to investigate the role of nervous system components such as neurotransmitter receptors (Mundy et al., 2021; Wang et al., 2023). Most of these studies used the swimming distance, sometimes combined with other parameters, as a behavioral endpoint. There was considerable variation in the developmental age of the fish (ranging from 3 dpf to 9 dpf), in the length of a light or dark phase (ranging from 2 min to 40 min), or the number of phase transitions (ranging from 1 to 5).

Several studies have looked at ways to improve the LDT or the subsequent data analysis. For example, Haigis et al. (2022) analyzed not only the average values of parameters (time and distance moved) but also additional parameters like the minimum and maximum. Hillman et al. (2024) assessed optimization of the light/dark phase length and the relevance of an acclimatization period. Two studies by the US EPA describe expanding the number of parameters to include, for example, habituation during the light or dark phase (Knapp et al., 2025; Rowson et al., 2025). Finally, Konrad et al. (2026) used LDT data to perform statistical power calculations, recommending 32 embryos per group.

We explored the possibilities of using AI/ML methods for analyzing zebrafish light-dark transition test data. For this purpose, we used four different methods (i.e., GLM, RF, GBM, and DL) that differ in nature and complexity (Orcales et al., 2024). Our study was not intended as a comprehensive method comparison, and therefore, other potentially useful algorithms were not included. The performance of AI/ML tools not only depends on the algorithm and the data set, but also on factors such as parameter settings and the kind of (cross-)validation. For this reason, we used the AI/ML functions available in a single h2o package, so that most of the data processing, including cross-validation and other settings, remains the same for each method (H2o.Ai, 2025). In preparing our data set, we used the 13 parameters available in the ZebraBox system. Rather than averaging these per light or dark phase, we kept the 1-min values for these parameters, thus keeping information related to factors such as variation within a light or dark phase, or habituation over time. This would be in line with proposals in recent studies (Haigis et al., 2022; Knapp et al., 2025; Rowson et al., 2025).

Overall, both univariate and AI/ML methods were able to distinguish groups for which we knew that a DNT effect should be present, for example, the continuously exposed groups on day 14. For several of the other groups studied, we did not identify any significant univariate parameters, but we were able to distinguish control and pharmaceutical-treated groups using AI/ML methods. This happens most clearly for fluoxetine after 5 days of continuous exposure, as well as on day 14 after exposure from day 0 to day 5. Given that we used n = 36 embryos per group and considering the recommendations by Konrad et al. (2026), it is not likely that our study was underpowered for univariate analysis. Rather, it indicates that AI/ML can outperform univariate statistics in detecting DNT effects in the LDT.

For three of the pharmaceuticals used (fluoxetine, paroxetine, and phenytoin), the exposed groups could already be distinguished from the control group after 5 days of exposure. This indicates that the window of vulnerability for these pharmaceuticals starts before 5 dpf. Although we should take care not to overgeneralize our conclusions, being able to distinguish DNT effects after 5 days instead of 14 days of exposure could be relevant from an animal welfare regulatory point of view, because zebrafish embryos until 120 h post-fertilization are not considered experimental animals under European legislation (European Commission, 2010; Strahle et al., 2012). For carbamazepine, effects after 5 days of exposure were not sufficiently strong for class discrimination. There are various possible explanations for why carbamazepine does not show a significant effect on day 5 compared to later timepoints, such as the concentration used or the developmental window of vulnerability, but resolving this is beyond the scope of this study.

In addition to detecting pharmaceutical-induced effects at early timepoints, AI/ML tools were also able to distinguish between reversible effects of anticonvulsants, which act by blocking voltage-gated sodium channels (Ferrendelli, 1987), versus irreversible effects of the antidepressants that act by selective serotonin reuptake inhibition (Leonard, 1995). The details of these effects and their relevance for human health protection will be discussed in another article (Hessel et al., in preparation).

As DMSO has been reported to potentially confound outcomes of the ZFET (Hoyberghs et al., 2021) and behavior assays (Chen et al., 2011; Huang et al., 2018; Christou et al., 2020), we performed a concentration–response analysis in both assays using DMSO up to 1%. The results demonstrated that, under the conditions in our laboratory (zebrafish strain and medium), no effect was observed up to a DMSO concentration of 1% (data not shown). This indicates that the results are unlikely to be confounded by a solvent-induced effect.

For the effects described in the previous paragraph, the GLM, RF, and DL methods showed similar performance, with GBM giving generally lower accuracies, although still distinguishing most exposures from controls. This ran contrary to our expectation that more advanced algorithms like GBM and DL might give better predictive performance. Because our study only uses a single data set, and because some AI applications (e.g., image processing or speech recognition) have benefitted from more advanced algorithms such as GBM and DL (Natekin and Knoll, 2013; LeCun et al., 2015), it is not possible to give reasons for the (dis)similarity of the performance of DL and GBM to the other two methods. However, it does indicate that, when implementing AI/ML methods in LDT analysis, it can be valuable to compare several algorithms before deciding on future implementations. A definite choice for an algorithm can then consider predictive performance, as well as factors such as computational speed and method interpretability, both of which would favor GLM and RF over GBM and DL. Among other factors, interpretability and transparency of AI/ML methods are important for risk assessors to have trust in these methods and establish regulatory acceptance of AI/ML models (Wassenaar et al., 2024; Hartung and Kleinstreuer, 2025; Gant et al., 2026).

In addition to pharmaceutical-exposed versus control comparisons, we also tested the method performance on random(ized) data. Partially, this served to verify if the performance on random(ized) data agreed with the intuitively expected value of 50%. Although this may seem self-evident, in some AI studies, a phenomenon known as data leakage, broadly defined as the illicit sharing of information between the training data and the test data, leads to strongly overestimated performances during model development (Bernett et al., 2024; Cahyana and Jang, 2025). This might occur, for example, if proper cross-validation is not performed, or if the data set used includes features such as a zebrafish ID number or well position that accidentally provides the model with information about what is to be predicted. By applying the AI/ML methods to random noise or label-permutated data, we confirmed that the random background accuracy is indeed close to 50%. In addition to this, we determined the variation in performance on random noise or label-permutated data to set a threshold for the distinction of actual effects. This would be useful, for example, in dose–response studies. Here, the variation for label-permutated data was larger than for random noise; hence, the former method would provide a more reliable threshold that the effect of substance exposure in the LDT exceeds background variation. If we set this as the upper limit of the 95% confidence interval, this would correspond to an accuracy in the range of 55.8%–61.1%, depending on the algorithm. With this in mind, we could conclude that the effects of carbamazepine after 5 days of continuous exposure or after 14 days with a short time exposure are not significantly different from random data and that the effects of phenytoin at 14 days after 5 days or 10 days of exposure should be interpreted with caution. Future studies may explore additional algorithms to assess method sensitivity.

The AI/ML tools were also used to compare the 14 dpf-unexposed control groups for the various pharmaceuticals. In principle, one should not expect any meaningful effects here. However, for practical logistical reasons, these studies were conducted over a period of 6 months, and therefore, control groups were formed using eggs from different spawning batches. We found that AI/ML tools were able to distinguish control groups. This indicates a need for (a) well-matched control group(s) in this kind of study.

In conclusion, we showed that AI/ML methods can be used to detect DNT in the zebrafish LDT. At least three of the AI methods tested appear more sensitive for detecting DNT than univariate statistical analysis. A potential drawback is that, by combining all parameters into a single analysis and single output value (i.e., accuracy), the gain in sensitivity to detect DNT effects comes at the expense of detail about which parameter(s) are affected, and therefore, insights into possible modes of action. AI/ML analyses should therefore not be used on their own but rather as part of a more comprehensive LDT data analysis. Additional studies are needed to further evaluate the strengths and weaknesses of AI/ML tools. Such future studies might also look in more detail at various timepoints to help further define the window of vulnerability for different compounds (Rice and Barone, 2000; Ingber and Pohl, 2016; Heyer and Meredith, 2017), gaining insights into differences between short-term and long-term effect (Ricarte et al., 2024) or complex dose–response relationships (Fong and Ford, 2014; van der Most et al., 2024). Ultimately, this will help to establish a best-practice workflow for the use of AI/ML in chemical risk assessment.

## Data Availability

The raw data supporting the conclusions of this article will be made available by the authors, without undue reservation.
